# Discovery of indole analogues from *Periplaneta americana* extract and their activities on cell proliferation and recovery of ulcerative colitis in mice

**DOI:** 10.3389/fphar.2023.1282545

**Published:** 2023-10-20

**Authors:** Yuchen Xie, Siwei Liang, Yifan Zhang, Taoqing Wu, Yongmei Shen, Shun Yao, Jing Li

**Affiliations:** ^1^ Key Laboratory of Bio-Resources and Eco-Environment (Ministry of Education), College of Life Sciences, Sichuan University, Chengdu, China; ^2^ School of Chemical Engineering, Sichuan University, Chengdu, China; ^3^ Sichuan Key Laboratory of Medical American Cockroach, Chengdu, China

**Keywords:** medicinal insects, indole analogues, *Periplaneta americana* extract, bioactivities, anti-ulcerative colitis

## Abstract

**Background:** As an important medicinal insect, *Periplaneta americana* (PA) has been applied for the treatment of wounds, burns, and ulcers with fewer side effects and a reduced recurrence rate, which provides great potential for developing new drugs based on its active constituents.

**Materials and methods:** The main chromatographic peaks determined by high performance liquid chromatography (HPLC) in the PA concentrated ethanol-extract liquid (PACEL) were separated, purified, and identified by semi-preparative LC, mass spectrum, and ^1^H NMR spectroscopic analysis. The biological activities of the identified compounds were investigated by methylthiazolyldiphenyl-tetrazolium bromide (MTT) method based on *in vitro* human skin fibroblasts (HSF) and *in vivo* experiments based on dextran sulfate sodium (DSS)-induced ulcerative colitis (UC) mouse model. Furthermore, RT-qPCR of six genes related to inflammation or intestinal epithelial cell proliferation was employed to investigate the molecular mechanism of the indole analogues recovering UC in mice.

**Results:** Five indole analogues were purified and identified from PACEL, including tryptophan (Trp), tryptamine (pa01), 1,2,3,4-tetrahydrogen-β-carboline-3-carboxylic acid (pa02), (1S, 3S)-1-methyl-1,2,3,4-tetrahydrogen-β-carboline-3-carboxylic acid (pa03), and (1R, 3S)-1-methyl-1,2,3,4-tetrahydrogen-β-carboline-3-carboxylic acid (pa04), among which the pa02 and pa04 were reported in PA for the first time. *In vitro* and *in vivo* experiments showed that PACEL, Trp, and pa02 had promoting HSF proliferation activity and intragastric administration of them could alleviate symptoms of weight loss and colon length shortening in the UC mice. Although recovery activity of the compound pa01 on the colon length was not as obvious as other compounds, it showed anti-inflammatory activity in histological analysis. In addition, The RT-qPCR results indicated that the three indole analogues could alleviate DSS-induced intestinal inflammation in mice by inhibiting pro-inflammatory cytokines (*MMP7*, *IL1α*) and down-regulating *BMP8B* expression.

**Conclusion:** This study reported the isolation, purification, structure identification, and biological activity of the active indole analogues in PACEL. It was found for the first time that the PA extract contained many indole analogues and Trp, which exhibited good proliferation activity on HSF fibroblasts as well as anti-UC activity in mice. These indole analogues probably are important components related to the pharmacological activity in PA.

## 1 Introduction

Recently, traditional Chinese medicine and natural medicine have been found effective in the treatment of many chronic diseases such as ulcerative colitis (UC), with fewer side effects and a reduced recurrence rate (Boal Carvalho and Cotter, 2017; Liu et al., 2022). UC can cause inflammation in the rectum, colon mucosa, and submucosa of the colon (Miller et al., 2016; Sarvestani et al., 2021). At present, the treatment of UC is mainly based on glucocorticoids, biological agents, and immunosuppressants. Many adverse reactions can occur as a result of treatment with 5-aminosalicylate or corticosteroids, including fever, kidney damage, edema, headaches, etc (Yokoyama et al., 2014; Ye et al., 2015). Identifying the therapeutic agents that can improve the process of intestinal healing from traditional Chinese medicines is of significance for treating UC, especially from those featured animals.

*Periplaneta americana* (PA) is an important medicinal insect, which was first published in Shennong’s Classic of Meteria Medica, and has been applied for the treatment of wounds, burns, ulcers, and primary liver cancer (Zeng et al., 2019). In recent years, continuous studies on PA show that the PA extract possesses many biological activities, including anti-tumor (Zhao et al., 2017), wound-healing (Liang et al., 2022), anti-liver fibrosis (Li et al., 2018), and antioxidant (Yang et al., 2022), anti-inflammatory (Lu et al., 2019), and anti-bacterial activity (Ali et al., 2017). Compared with these biological activity researches, the chemical studies on the pharmacodynamic active ingredients in PA lag behind. At present, the reported ingredients from PA include amino acids, peptides, lipids, nucleosides, alkaloids, flavonoids, polysaccharides, etc (Kim et al., 2016; Zhao et al., 2022). The research on the biological activity of the complex extract can become the guide for the following separation of a series of target components. For example, several biologically active compounds were successfully identified from the extract of PA by taking the wound repair effect as a guide (Machado et al., 2010). Previous studies indicated that the extract of PA can promote the growth of new granulation tissue, which can be used for gastric ulcers (Lu et al., 2019; Fu et al., 2021). However, the pharmacodynamic active ingredients in PA have not been completely identified and confirmed so far.

β-Carboline is a group of indole alkaloids with the important representative compounds of tryptoline, pinoline, harmane, harmine, harmaline, and tetrahydroharmine (Bratchkova et al., 2012). Based on current researches, a wide spectrum of pharmacological and psychopharmacological activities of β-carbolines are of great interest including vasorelaxant (Shi et al., 2001), antidepressant (Aricioglu and Altunbas, 2003; Herraiz and Chaparro, 2005), antiparasitic (Di Giorgio et al., 2004), antitumor (Zhang et al., 2016), antimicrobial (Suzuki et al., 2018), antiviral (Ashok et al., 2015) and antioxidant (Herraiz and Galisteo, 2002) activities. Particularly, the β-carboline alkaloids were widely distributed in some herbal plants, i.e., *Peganum harmala* (Zygophillaceae, Syrian Rue) (Cao et al., 2007), yacon leaves (Yuan et al., 2017) and *Passiflora alata* leaves (Zhu et al., 2018), while the existence of these compounds in animals, especially insects is worthy exploring.

In this study, several active chemical components of indole analogues were isolated and identified from the PA extract, and the biological activities of the identified compounds were verified at the cellular, *in vivo*, and molecular levels. The main five chromatographic peaks which were determined by high performance liquid chromatography (HPLC) in the extract of PA were separated, purified, and identified. Based on the PA extract and three of the identified indole analogues, we evaluated both *in vitro* proliferation activity on human skin fibroblasts (HSF) as well as *in vivo* activities on recovery of UC induced by dextran sulfate sodium (DSS) in mice. Furthermore, RT-qPCR was employed to investigate the effects of PA extract and three compounds on the gene expression of six genes related to inflammation or intestinal epithelial cell proliferation in UC mice. The whole results are expected to lay the foundation for the further research and development of PA.

## 2 Materials and methods

### 2.1 Materials and reagents

The PA concentrated ethanol-extract liquid (PACEL) (containing 2 g worms/mL) was provided by Good Doctor Company (Chengdu, Sichuan province, China) for HPLC analysis and separation, and was also used for experiments on proliferation-promoting activity and effects on UC in mice. HSF cells were incubated in a 37°C cell incubator filled with 5% CO_2_. The 8-week-old SPF-grade ICR male mice were purchased from Dossy Co., Ltd. (Chengdu, Sichuan province, China), and fed in the IVC ventilation cages of the SPF animal lab at the College of Life Sciences of Sichuan University according to the standard procedures of animal experiments. The lab temperature was kept at 22°C–24°C, and the free-feeding mice were exposed to 12 h:12 h light/dark cycle light. The drinking water utensils and cages were cleaned regularly. The conventional sterilization and maintenance of mouse feed (Dossy Co., Ltd., Chengdu, Sichuan province, China) was provided. Ultrapure water was obtained by a water purification system UPH-1-10T (ULUPURE, Chengdu, Sichuan province, China).

Dulbecco’s Modified Eagle’s Medium (DMEM) and fetal bovine serum (FBS) were obtained from Gibco Company (Paisley, United Kingdom). Thiazolyl blue tetrazolium bromide (MTT) and dimethyl sulfoxide (DMSO) were purchased from Sigma Chemical Co. (St. Louis, MO, United States). L-tryptophan (Trp) and tryptamine (pa01) were purchased from Shenggong Bioengineering Technology Service Co., Ltd. (Shanghai, China), and 1,2,3,4-tetrahydronorharman-3-carboxylic acid (pa02) were purchased from McLean Biochemistry Co., Ltd. (Shanghai, China). Mei5 Biotechnology Co., Ltd. (Beijing, China) supplied the M5 Total RNA Extraction Reagent (TRIgent). DSS (molecular weight 36,000–50,000), Hifair ®III 1st Strand cDNA Synthesis Kit (gDNA digester plus), Hieff UNICON ®universal Blue qPCR SYBR Green Master Mix were purchased from Yeasen Biotechnology Co., Ltd. (Shanghai, China).

### 2.2 Equipments

Ultrapure water meter, ultra-clean workbench, centrifuge, CO_2_ incubator, and automatic enzyme analyzer were provided by Thermo Scientific, United States. The ordinary optical microscope and inverted microscope were purchased from Olympus, Japan. The rotary evaporator was purchased from Yarong Co., Ltd. (Shanghai, China). The freeze dryer was purchased from Zhengqiao Co., Ltd. (Shanghai, China). Wisleap WS-10 digital panoramic scanner (non-fluorescent) (WISLEAP medical technology Co., Ltd., Jiangsu, China) was used for the analysis of biological tissue slice images. Multiskan GO continuous wavelength enzyme marker (Thermo Fischer Scientific, Vantaa, Finland) was used for absorbance detection. Mass spectrometry was performed on Waters Q-TOF Premier (Waters MS Technologies, Manchester, United Kingdom) and ^1^H-NMR spectra were recorded on Bruker AV Ⅱ-600 MHz spectrometer (Bruker, Bremen, Germany).

### 2.3 HPLC analysis and separation of PACEL

After removing the solvent of the PACEL (300 mL) under vacuum, ultrapure water (600 mL) was used to prepare the PA solution (PAS) for further HPLC analysis. Chromatographic analysis was performed on a 1260 HPLC infinity system (Agilent, United States) equipped with an Agilent ZORBAX SB-C_18_ column (4.6 × 150 mm, 5 μm). The mobile phase consisted of solvent A (acetonitrile) and B (0.1% trifluoroacetic acid in water), with an injection volume of 10 μL. The column temperature was 30°C, and a DAD detector was applied with an ultraviolet wavelength of 220 nm. A flow rate of 0.1 mL/min with a linear gradient was used as follows: 0–30 min, 15%-35% A.

After HPLC analysis, the target peaks were prepared by semi-preparative liquid chromatography with a Chromatorex C_18_ column (10 × 250 mm, 10 μm). The mobile phase was 18.5% acetonitrile (containing 0.07% trifluoroacetic acid). The injection volume was 1 mL, the flow rate was 2.5 mL/min, and the detection wavelength was 220 nm. The solution of the target peak was manually collected at the corresponding peak time, which was concentrated and then freeze-dried. The products were further purified by Sephadex LH-20 gel (50-150 μm) column chromatography. Finally, the chromatograms, mass spectral data, and ^1^H-NMR were compared with that of the reference data, and the molecular weight, formula, and structure of the compounds were determined through the SciFinder database.

Due to the low content of pa01, the purification process of the chromatographic peak component was different from the above method. Before the separation and analysis of HPLC, the crude extract of pa01 was prepared through preparative silica G thin layer plates (Ocean Chemical Inc., Qingdao, China) with the developing agent of chloroform: methanol: n-butanol: glacial acetic acid = 3.1: 0.9: 0.5: 0.5 (V/V/V/V) and then purified by Sephadex LH-20 gel (50-150 μm) column.

### 2.4 Proliferation-promoting activity of PACEL and three indole analogues on HSF cells

HSF cells in the logarithmic growth phase were used for testing the proliferation-promoting activity of PACEL and three indole analogues, including Trp, pa01, and pa02. Due to lack enough sample of pa03 and pa04, they were not included for the bioactivity investigation. The blown cell suspension was transferred and centrifuged at 1,500 rpm for 3 min at room temperature. DMEM medium was added to re-suspend the HSF cells, then added in a 96-well cell plate at a concentration of 5 × 10^3^ cells/mL. The proliferation-promoting activity was examined by the MTT method (Shi et al., 2019). PACEL was diluted with ddH_2_O and added to a 96-well cell plate at a final concentration of 0.1, 0.5, 1, 5, 10, 20, 50, 100 mg/mL in each well. Trp and pa02 were diluted with ddH_2_O and added in the cell plate at the final concentration in each well of 0.01, 0.05, 0.1, 0.5, 1, 5, 10, 50 μg/mL respectively. Besides, pa01 was dissolved in 40% ethanol and added to the cell plate with a final concentration of 1, 2, 5, 10, 20, 50, 100, 250 μg/mL in each well; and four repeats were set for each concentration. The negative control wells (adding an equal amount of solvent) and the blank wells (without adding) were set up at the same time. The 96-well plate was incubated at 37°C in a 5% CO_2_ cell incubator for 24 h, then methylthiazolyldiphenyl-tetrazolium bromide (MTT, 20 μL/well) was added for further culture for 4 h. After adding 150 μL of DMSO per well, the absorbance value (OD value) was measured with an automatic enzyme analyzer at a wavelength of 570 nm. The average value of four wells in each group was calculated.

### 2.5 Effects of PACEL and three indole analogues on ulcerative colitis model mice

Firstly, the acute UC model of mice was induced by sodium dextran sulfate (DSS) (Ge et al., 2021). DSS was dissolved in ddH_2_O to prepare 4% DSS (W/W) solution every day. It was provided to experimental mice instead of drinking water for 8 days except for the control group; during this time, mice were allowed to voluntarily ingest standard animal feed. Secondly, after successfully establishing the acute colitis model in mice, they were randomly divided into five groups: the PACEL, the Trp, the pa01, and the pa02 treatment groups together with the model group. Each group included five mice, and they were given intragastric administration of PACEL, indole analogues, or ddH_2_O once a day. Mice in the control group were given ddH_2_O during the 16 days, the model group was given ddH_2_O from 9th to 16th day, while the other four groups were given PACEL (2 g/kg BW), Trp (100 mg/kg BW), pa01 (200 mg/kg BW), or pa02 (10 mg/kg BW) from 9th to 16th day.

In the process of modeling, mice in the control group were observed with smooth shiny hair, good activity, and no decrease in body weight. Whereas, the DSS-ingested mice showed reduced food intake, serious weight loss, poor mental state, sparse dull hair, and so forth. In addition, they developed feces from a state of soft and shapeless to bloody stool, indicating the success of inducing the acute UC model (Ge et al., 2021). During the 16 days, the change in body weight of mice was recorded and the rate of body weight (%) could be obtained as the ratio of weight in the *n*th day to initial body weight. At the same time, mortality, food intake, water intake, fecal morphology, and hematochezia were observed every day (Kim et al., 2012). On the 9^th^ day, four experimental mice were randomly selected and euthanized to examine the efficiency of the model induced. On the 16th day, all the mice were executed by cervical dislocation and dissected. After the mice were euthanized, the length of the large intestine was measured, and the appearance of intestinal mucosal was observed and recorded. The colon tissues were cut to prepare paraffin sections, and fixed with 4% paraformaldehyde, dehydrated by ethanol gradient, and embedded in paraffin. The tissue sections were cut at a thickness of 5 μm and stained with hematoxylin-eosin (HE), and the morphological and pathological changes were observed under the light microscope (Li et al., 2020). The digital panoramic scanner (non-fluorescent) and WisleapWS-10 image analysis system were used to analyze the picture.

### 2.6 Effects of PACEL and three indole analogues on gene expression in mice with ulcerative colitis

By collecting fresh mouse colonic tissue, the total RNA was extracted by the M5 Total RNA Extraction Reagent (TRIgent) kit. Hifair ®III 1st Strand cDNA Synthesis Kit (gDNA digester plus) kit was used for reverse transcription of the total RNA. 15 μL mixed liquid was mixed in the RNase-free centrifuge tube, including 10 μL RNase-free H_2_O, 3 μL 5 × gDNA Digester Mix, and 2 μL RNA. The mixture was gently blown with a pipette and incubated at 42°C for 2 min. Subsequently, 10 × Hifair ®III Super Buffer 2 μL, Hifair ®III RT Enzyme Mix 1 μL, Random Primers N6 and Oligo (dT) 18 1:1 mixture 1 μL, RNase-free H_2_O 1 μL were added to gently blow and mix. The program was setting as following: 25°C, 5 min; 55°C, 15 min; 85°C, 5 min, and the product was stored at −80°C.

To examine the effect of PACEL and the indole analogues on gene expression in mice with UC, six genes related to inflammation, immunity, or intestinal epithelial cell proliferation were selected for RT-qPCR with *GAPDH* as the internal control. Primers (Table 1) were designed in the conservative region using Primer-BLAST (http://www.ncbi.nlm.nih.gov/tools/primer-blast) and were synthesized by Shenggong Biological Engineering Technology & Services Co., Ltd. (Shanghai, China). Real-time fluorescence quantitative PCR reaction system: Hieff UNICON^®^ universal Blue qPCR SYBR Green Master Mix 10 μL, Forward Primer (10 μM) 0.4 μL, Reverse Primer (10 μM) 0.4 μL, cDNA 1 μL, ddH_2_O 8.2 μL. Program settings were as follows: pre-denaturation 95°C, 2 min; denaturation 95°C, 10 s; annealing/extension 60°C, 30 s; amplification 40 cycles; melting curve stage, instrument default setting. The mean value of the cycle threshold (Ct value) was calculated, and then the relative content of each gene in tissue was analyzed by comparison 2^−ΔΔCt^ method (Li et al., 2020). Finally, GraphPad (Prism 8.0.2) was applied for data statistics and one-way ANOVA.

**TABLE 1:** SYBR Green real-time qPCR primer sequences.

Primer	Forward	Reverse
*MMP7*	CTG​CCA​CTG​TCC​CAG​GAA​G	GGG​AGA​GTT​TTC​CAG​TCA​TGG
*IL1α*	CGA​AGA​CTA​CAG​TTC​TGC​CAT​T	GAC​GTT​TCA​GAG​GTT​CTC​AGA​G
*MUC4*	GAG​AGT​TCC​CTG​GCT​GTG​TC	GGA​CAT​GGG​TGT​CTG​TGT​TG
*BMP8B*	CCG​GGA​CTC​CTA​TGG​CTA​CT	CAT​CCG​TCA​TGG​CAC​GGT​A
*AGT*	TCT​CCT​TTA​CCA​CAA​CAA​GAG​CA	CTT​CTC​ATT​CAC​AGG​GGA​GGT
*PTGS2*	GCT​CAG​CCA​GGC​AGC​AAA​TC	ACC​ATA​GAA​TCC​AGT​CCG​GGT
*GAPDH*	ATG​GGA​AGC​TTG​TCA​TCA​ACG	AAG​ACA​CCA​GTA​GAC​TCC​ACG

## 3 Results and discussions

### 3.1 HPLC analysis and separation of PACEL

**FIGURE 1:**
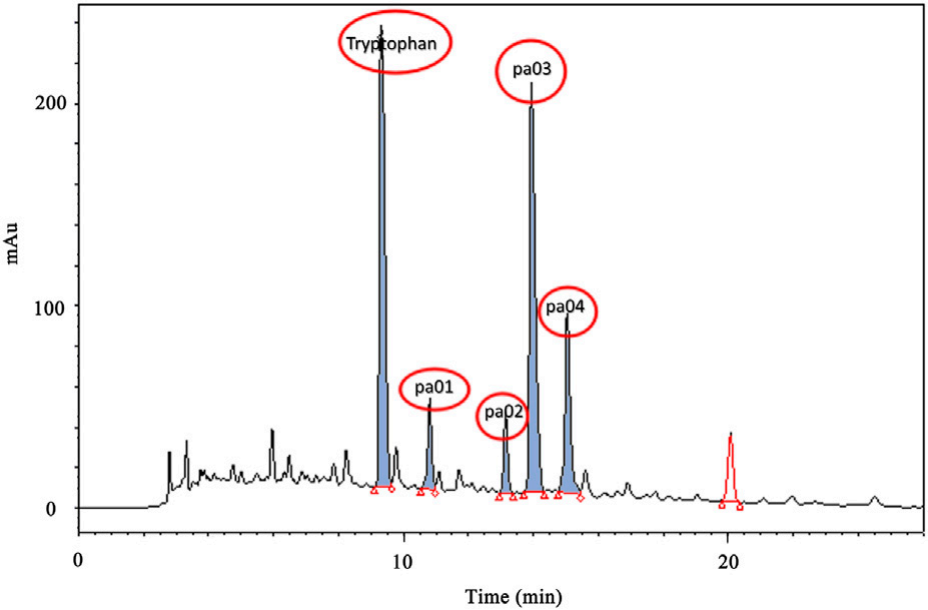
HPLC gradient elution image of PA extract.

After concentration under vacuum, the PACEL was dissolved in ultra-pure water and qualitatively filtered, from which PA solution (PAS) was obtained. Then HPLC was used for gradient elution of PAS. As shown in Figure 1, the five components with high content in the PAS were concentrated between 8 min and 16 min. The chromatographic peak with the highest content at the retention time of 6.834 min was determined as Trp, which was consistent with the peak time of the standard compound of Trp. The other four chromatographic peaks with relatively high content are named pa01 (8.52 min), pa02 (11.68 min), pa03 (13.21 min), and pa04 (15.46 min) respectively.

After the separation and purification of components at the four peaks by semi-preparative high-performance liquid chromatography and column chromatography, the high purity of the four components was obtained (above 98.0%). By integrating the results in mass spectra and ^1^H NMR (Supplementary Figures S1, S2), the compounds corresponding to peaks pa01, pa02, pa03, and pa04 were successfully identified as follows:

*2-*(*1H-idole-3-yl*)*ethylamine* (pa01, Tryptamine, CAS: 61-54-1): ^1^H NMR (d_6_-DMSO, 600 MHz) δ: 10.99 (s, 1H), 7.65 (s, 1H), 7.55 (d, J = 7.8 Hz, 1H), 7.37 (d, J = 8.1 Hz, 1H), 7.23 (d, J = 2.2 Hz, 1H), 7.09 (t, J = 7.5 Hz, 1H), 7.01 (t, J = 7.4 Hz, 1H), 3.08 (dd, J = 8.9, 6.6 Hz, 2H), 2.99 (dd, J = 8.8, 6.6 Hz, 2H); ^13^C-NMR (d_6_-DMSO, 600 MHz) δ: 23.5 (C-β), 39.7 (C-α), 109.8 (C-3), 112.0 (C-7), 118.9 (C-4), 121.6 (C-6), 123.9 (C-5), 127.3 (C-2), 136.8 (C-3a), 158.5 (C-7a); TOF-MS: m/z: 161.1068 [M+H]^+^.

*1,2,3,4-Tetrahydrogen-β-carboline-3-carboxylic acid* (pa02, CAS: 6052-68-2): ^1^H NMR (d_6_-DMSO, 600 MHz) δ: 10.91 (s, 1H), 7.42 (d, J = 7.8 Hz, 1H), 7.31 (d, J = 8.1 Hz, 1H), 7.05 (t, J = 7.7 Hz, 1H), 6.97 (d, J = 7.5 Hz, 1H), 4.21 (d, J = 15.6 Hz, 1H), 4.14 (d, J = 15.4 Hz, 1H), 3.57 (dd, J = 10.6, 4.8 Hz, 2H), 3.11 (dd, J = 15.9, 4.9 Hz, 2H), 2.78 (dd, J = 15.9, 10.6 Hz, 1H); TOF-MS: m/z 217.0991 [M+H]^+^.

*(1S, 3S)-1-methyl-1,2,3,4-tetrahydrogen-β-carboline-3-carboxylic acid* (pa03, CAS: 5470-37-1): ^1^H NMR (d_6_-DMSO, 600 MHz) δ: 11.06 (s, 1H), 7.46 (d, J = 7.8 Hz, 1H), 7.34 (d, J = 8.1 Hz, 1H), 7.1–7.07 (m, 1H), 7.01 (t, J = 7.4 Hz, 1H), 4.53 (d, J = 7.0 Hz, 1H), 3.68 (dd, J = 12.0, 4.8 Hz, 2H), 3.21–3.15 (m, 2H), 2.83–2.75 (m, 1H), 1.62 (d, J = 6.7 Hz, 3H); TOF-MS: m/z 231.1157 [M+H]^+^.

*(1R, 3S)-1-methyl-1,2,3,4-tetrahydrogen-β-carboline-3-carboxylic acid* (pa04, CAS: 5470-37-1): ^1^H NMR (d_6_-DMSO, 600 MHz) δ: 10.93 (s, 1H), 7.42 (d, J = 8.0 Hz, 1H), 7.31 (d, J = 8.1 Hz, 1H), 7.07 (t, J = 7.5 Hz, 1H), 6.98 (t, J = 7.4 Hz, 1H), 4.58 (q, J = 6.9 Hz, 1H), 3.73 (dd, J = 8.0, 5.5 Hz, 2H), 3.05 (dd, J = 15.8, 5.4 Hz, 2H), 2.91 (dd, J = 15.8, 8.0 Hz, 1H), 1.54 (d, J = 6.8 Hz, 3H); TOF-MS: m/z 231.1145 [M+H]^+^.

**FIGURE 2:**
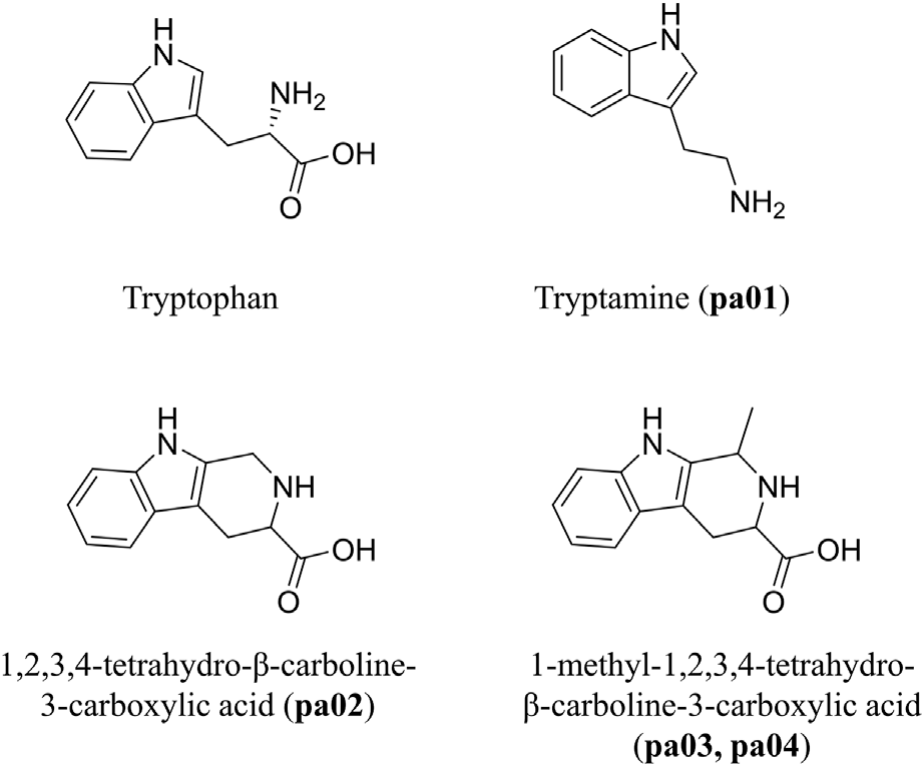
Structure of tryptophan, tryptamine, 1,2,3,4-tetrahydrogen-β-carboline-3-carboxylic acid, 1-methyl-1,2,3,4-tetrahydrogen-β-carboline-3-carboxylic acid.

The structural formula of the four components and Trp is shown in Figure 2. The identification results showed that the structures of pa01, pa02, pa03, and pa04 were similar to Trp and all of them contained indole rings, which indicates an intrinsic relationship in their biosynthesis. In particular, pa02, pa03, and pa04 were all β-carboline alkaloid compounds, containing tetrahydro-β-carboline structure, wherein pa03 and pa04 were isomers of each other, respectively 1S, 3S (pa03) and 1R, 3S (pa04) configurations.

### 3.2 Proliferation activity of PACEL and three indole analogues on the HSF cells

**FIGURE 3:**
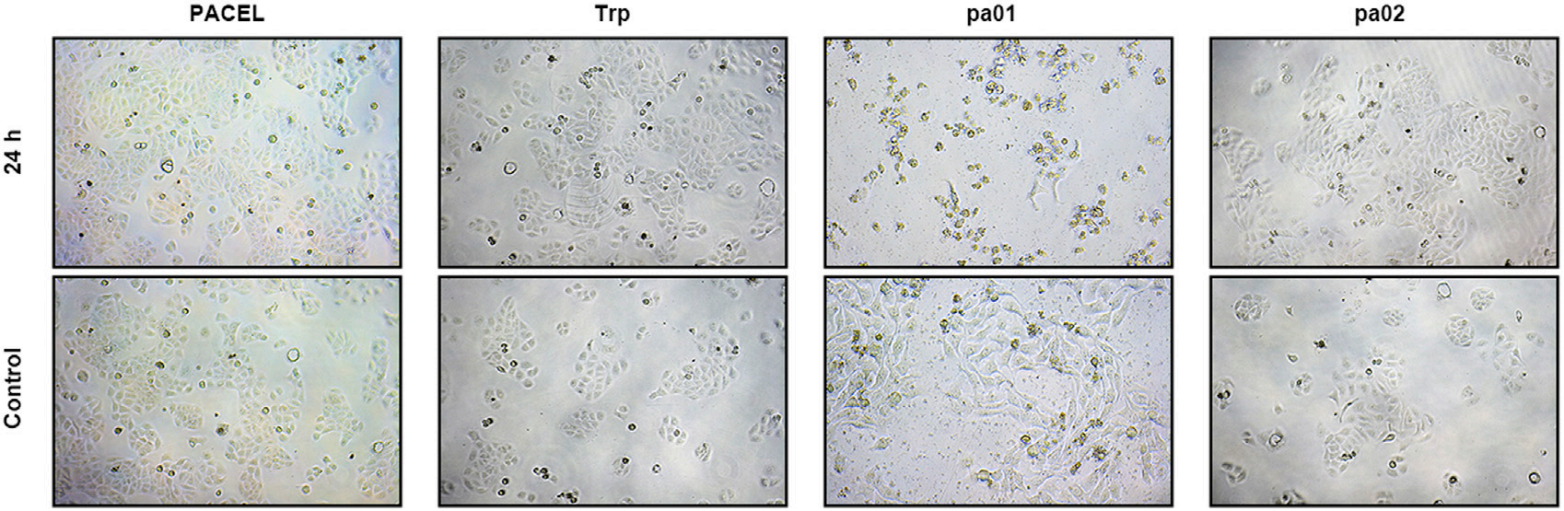
Effect of extract and three indole analogues from PA on HSF cell viability. Representative pictures of HSF treated with four drugs at different concentrations (PACEL 1 mg/mL, Trp 0.1 μg/mL, pa01 250 μg/mL, pa02 0.1 μg/mL).

The proliferation activity of PACEL and three indole analogues (Trp, pa01, and pa02) on HSF cells was evaluated by the MTT method. As the result, PACEL, Trp, and pa02 all exhibited proliferation-promoting activities, while pa01 showed an anti-proliferation activity (Table 2; Figure 3). Particularly, even at a low concentration of 0.1 μg/mL, the proliferation of HSF cells in Trp and pa02 group was significantly higher than that of the control group (*p* < 0.05). In contrast, pa01 inhibited the proliferation of HSF cells in a concentration-dependent manner with the IC_50_ of 62.14 μg/mL. When pa01 > 50 μg/mL, the HSF cell proliferation in the pa01 group was significantly lower than that of the control (*p* < 0.05). The anti-proliferation activity of pa01 might contribute to the decreased proliferation activity in high concentrations of PACEL (Table 2). Given that Trp and pa02 had relatively high content in PACEL compared with pa01, PACEL showed a proliferation-promoting activity at low concentrations; while at the high concentration of PACEL, the proliferation activity decreased due to the anti-proliferation activity of pa01.

**TABLE 2:** Effect of extract and three indole analogues from PA on HSF cell proliferation.

Groups	PACEL (24 h)		Trp (24 h)		pa01 (24 h)		pa02 (24 h)	
	C (mg/mL)	Absorbance value	C (μg/mL)	Absorbance value	C (μg/mL)	Absorbance value	C (μg/mL)	Absorbance value
Test groups	0.1	1.88 ± 0.05	0.01	0.89 ± 0.05	1	2.07 ± 0.19	0.01	0.95 ± 0.02
	0.5	1.96 ± 0.05	0.05	0.98 ± 0.05	2	2.27 ± 0.23	0.05	0.93 ± 0.04
	1	2.05 ± 0.06**	0.1	1.12 ± 0.02*	5	2.16 ± 0.10	0.1	0.96 ± 0.03*
	5	2.01 ± 0.08*	0.5	1.10 ± 0.02	10	2.05 ± 0.15	0.5	0.90 ± 0.01
	10	1.97 ± 0.04	1	1.07 ± 0.02	20	1.96 ± 0.06	1	0.82 ± 0.03
	20	1.96 ± 0.07	5	1.01 ± 0.02	50	1.81 ± 0.07*	5	0.79 ± 0.03
	50	1.87 ± 0.08	10	0.94 ± 0.03	100	0.15 ± 0.02**	10	0.93 ± 0.03
	100	1.68 ± 0.11	50	0.97 ± 0.02	250	0.11 ± 0.01**	50	0.93 ± 0.04
Control group		1.86 ± 0.03		1.04 ± 0.02		2.21 ± 0.16		0.90 ± 0.03

Statistical analysis was done with one-way analysis of variance (ANOVA) compared with the control group (x ± s, *n* = 4), *p < 0.05, **p < 0.01.

### 3.3 Bioactivity of PACEL and three indole analogues on mice with UC

#### 3.3.1 Effects on mice body weight, survival rate and colon length

**FIGURE 4:**
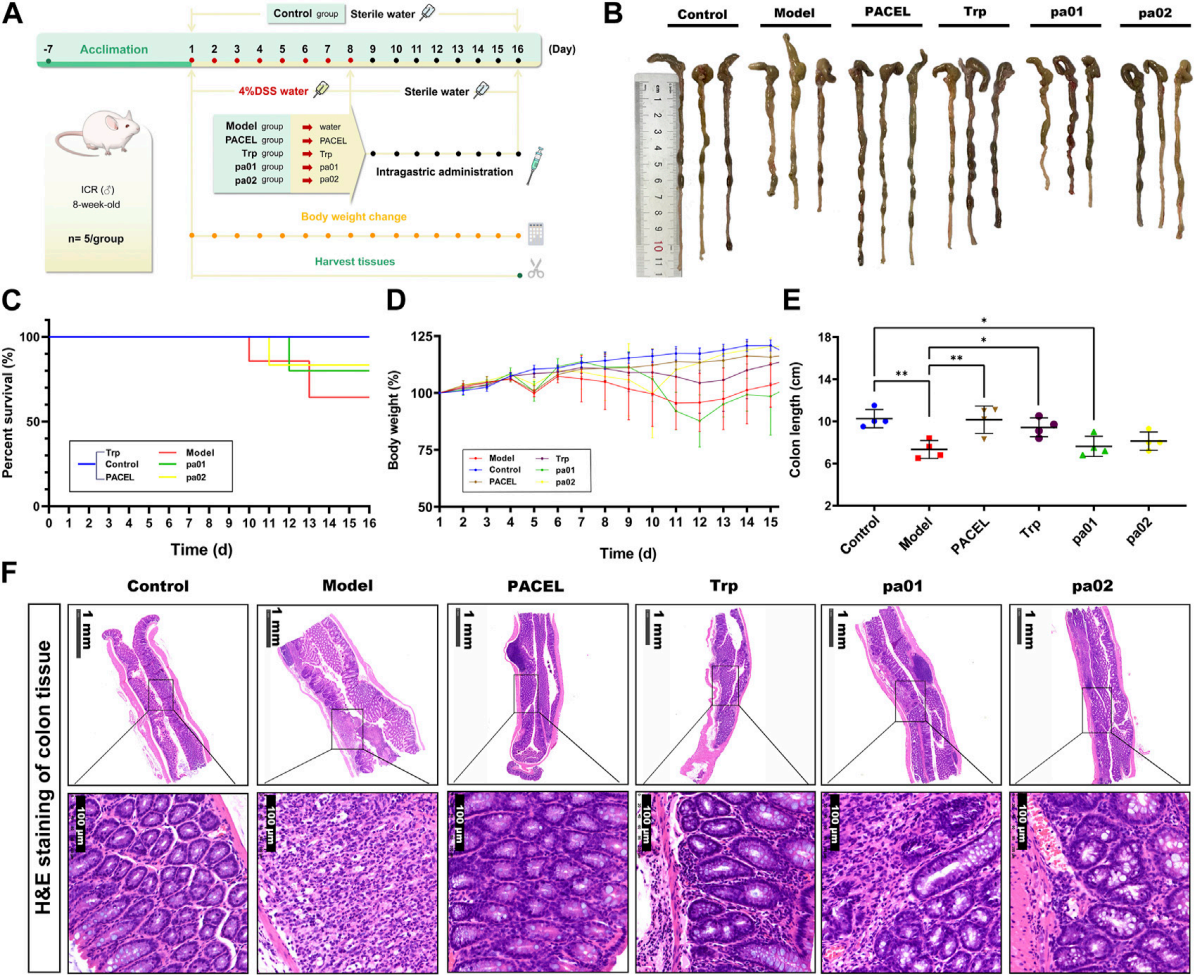
Comparison of the treatment effects of PACEL and the three indole analogues in DSS-induced UC mice. **(A)** Scheme of experimental procedure; **(B)** Representative colon images and the length of different groups; **(C)** Survival rate; **(D)** Change of body weight of mice in each group; **(E)** Colon length of each group; **(F)** Representative pictures of H&E stained colonic sections of mice in each group.

Based on the aforementioned operations (Figure 4A), the acute UC model was successfully established in mice induced by sodium dextran sulfate (DSS). Compared with other groups, mice in the model group showed obvious diarrhea symptoms excreting loose stool, even blood stool, with a mortality rate of 60%, while mortalities in other groups were zero or very low (Figure 4C). After being fed with PACEL, Trp, pa01, or pa02 to the UC model mice, the diarrhea symptoms were significantly improved, no yellow sticky stool was observed in the PACEL, Trp, or pa02 groups. Moreover, the body mass of mice in these groups recovered to some extent compared with the model group, with the highest recovery found in the pa02 group (Figure 4D). However, the pa01 treatment group showed no recovery in mice body weight although the mice mortality in the group was relatively low. At the end of the experiment, the mice were dissected and the colon length was examined (Figures 4B, E). Colon length in the control group was the longest, which was significantly longer than that in the model group (*p* < 0.01) and the pa01 group (*p* < 0.05), indicating minimal recovery effect of pa01 on mice colon. The PACEL, Trp, and pa02 all could recover the damages and regression of the colon induced by DSS. Mice after ingestion of Trp and pa02 had colon lengths significantly longer than the model group (*p* < 0.05 and *p* < 0.01), especially the PACEL group had colon lengths without significant difference from the control group (*p* > 0.05).

#### 3.3.2 Effect of PACEL and three indole analogues on colonic histology in UC mice

The effects of PACEL and three indole analogues on mice colonic histology were examined by the HE tissue section. It indicated that PACEL and all indole analogues had a repairing effect (Figure 4F). In the control group, there were notably intact crypts and goblet cells, and colon cells were clearly stained and arranged orderly; the glands were normal, and no inflammatory cells were observed. In the model group, mice colon exhibited severe inflammation, including epithelial erosion, interstitial oedema, and an increase in the number of inflammatory cells in the lamina propria. After treatment of PACEL, Trp, pa01, and pa02, symptoms of UC in the colon decreased at the histological level, including a particularly notable decrease in local lymphocytic infiltration and inflammatory cell infiltration. The results suggested that both PACEL and the three indole analogues could improve intestinal ulceration caused by DSS and restore the histological structure. In particular, the morphology of goblet cells was intact in the colon of the PACEL group and Trp group, indicating better recovery of them than pa01 and pa02.

#### 3.3.3 Effects of PACEL and three indole analogues on colonic gene expression in UC mice

**FIGURE 5:**
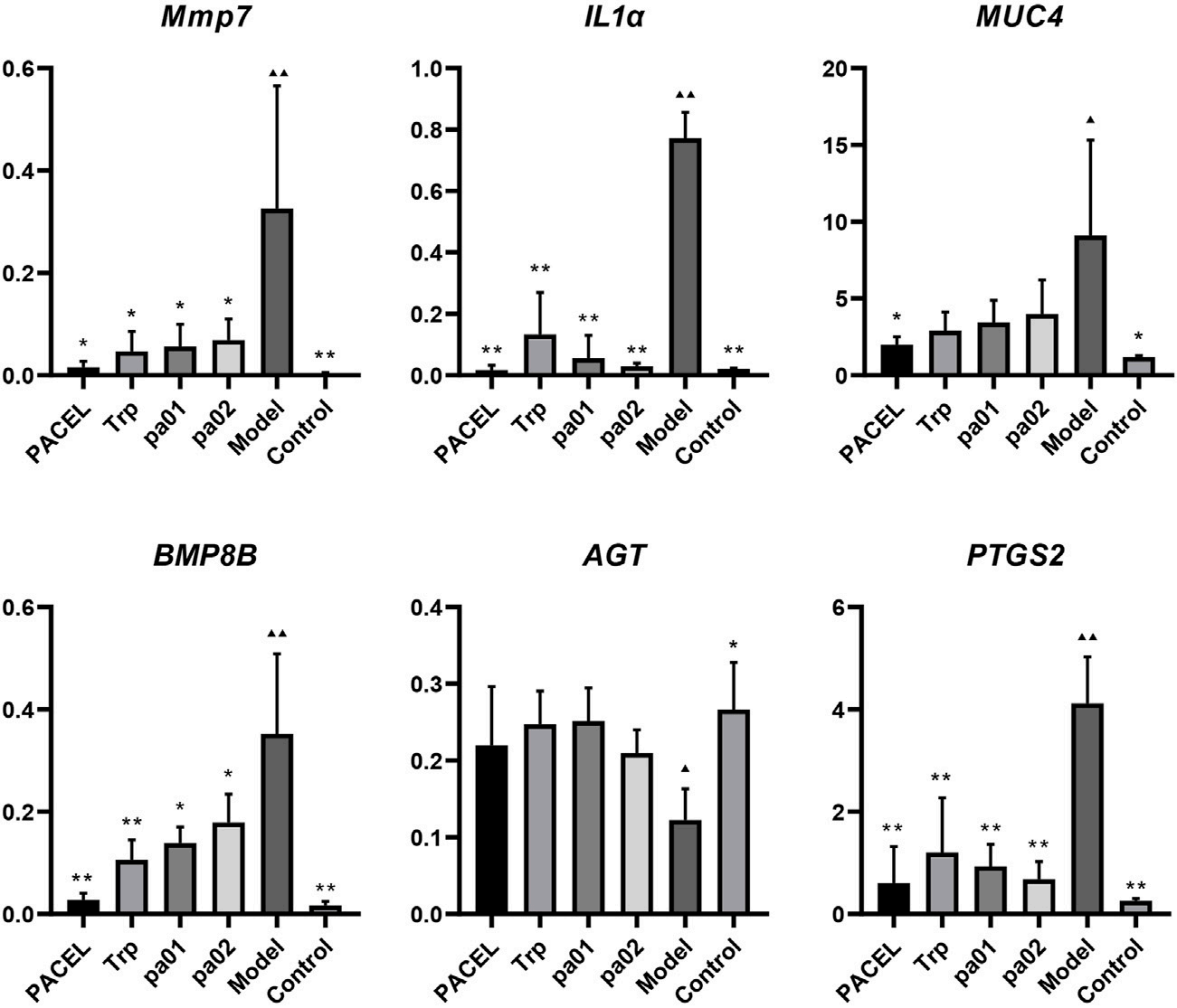
Key genes expression in colon tissues. **p < 0.001, *p < 0.05, statistical analysis was done with one-way analysis of variance (ANOVA), compared with the model group. ▲▲ *p* < 0.001, ▲ *p* < 0.05, compared with the control group.

Using the *GAPDH* gene as the internal standard, we employed RT-qPCR to investigate the effects of PACEL and three indole analogues on the gene expression of colon tissue of UC mice. As shown in Figure 5, gene expressions of the inflammatory cytokines-related genes (*MMP7*, *IL1α*), mucosal genes (*MUC4*, *BMP8B*), and one epithelial gene *PTGS2* were significantly increased, and expression of *AGT* gene was obviously decreased in the model group compared with the control group, indicating that severe barrier injury and inflammatory reaction occurred in the intestines of mice treated with DSS, and no recovery was found in the model group. However, gene expression levels in mice treated with PACEL, Trp, pa01, or pa02 showed no significant difference from the control group (*p* > 0.05), indicating that both PACEL and the three indole analogues could regulate the expression of inflammatory genes, mucosal genes, and epithelial genes in mice colon to a normal level. Compared with the model group, intragastric administration of PACEL significantly downregulated gene expression of *MMP7*, *IL1α*, *MUC4*, *BMP8B*, and *PTGS2* (*p* < 0.05), Trp or pa01 treatment significantly downregulated *MMP7*, *IL1α*, *BMP8B*, and *PTGS2* genes (*p* < 0.05), and pa02 treatment significantly downregulated *MMP7*, *IL1α*, and *PTGS2* genes (*p* < 0.05). While both PACEL and the three indole analogues significantly upregulated expression of the *AGT* gene (*p* < 0.05) and all of these showed an inhibiting inflammation activity and alleviated the damage of the intestinal barrier induced by DSS.

## 4 Discussion

Through the analysis by HPLC and separation by semi-preparative LC and column chromatography, five indole analogues in PACEL were successfully obtained and identified, including tryptophan (Trp), tryptamine (pa01), 1,2,3,4-tetrahydrogen-β-carboline-3-carboxylic acid (pa02), (1S, 3S)-1-methyl-1,2,3,4-tetrahydrogen-β-carboline-3-carboxylic acid (pa03), and (1R, 3S)-1-methyl-1,2,3,4-tetrahydrogen-β-carboline-3-carboxylic acid (pa04). It is important to note that pa02 and pa04 were first reported in PA, while pa03 has been reported in the previous study (Li et al., 2015), which was isolated from the ethyl acetate fraction of the ethanol extract of PA. Among the constituents, pa01 was an indolic derivative from Trp degradation and was widespread in foods and human organisms (Innocente et al., 2007; Campos Chiste et al., 2017; Zhang et al., 2019). Tryptamine is considered a neuromodulator or neurotransmitter (Khan and Nawaz, 2016) and has previously been characterized as an aryl hydrocarbon receptor (AHR) agonist (Vikström Bergander et al., 2012), which can activate AHR to regulate necrotic apoptosis and inflammation. The activation of AHR is crucial in regulating the intestinal barrier, and similar agonists have been extensively reported in the treatment of UC (Li et al., 2021; Peng et al., 2021). Particularly, pa02, pa03, and pa04 were identified as β-carbolines (a group of indole alkaloids) which have been reported mainly in plants such as yacon leaves (Cao et al., 2007) and *Portulaca oleracea* (Xiu et al., 2018) for a wide spectrum of pharmacological activities including vasorelaxant, antidepressant, antiparasitic, antimicrobial and antitumor activities. Biological activity studies have shown that Trp can improve free radical scavenging ability, increase superoxide dismutase activity and reduce lipid peroxidation (Yao et al., 2011). The ABTS radical scavenging experiment has also proved that β-Carboline alkaloids exhibited obvious antioxidant capacity with an electron transfer mechanism on the indole ring (Ashok et al., 2015). As a direct precursor of harman, 1-methyl-1,2,3,4-tetrahydrogen-β-carboline-3-carboxylic acid appeared as two diastereoisomers (1S, 3S and 1R, 3S) with the same mass spectra and high levels of these chemicals in raisins were identified as the key contributors to monoamine oxidase (MAO) inhibition (Herraiz, 2007). The first report of high levels of Trp and abundant β-carbolines in PA proved their significant bioactivities.

To investigate the bioactivities of the identified indole analogues, we examined both fibroblast proliferation activity *in vitro* and bioactivities of PACEL and three indole analogues in UC model mice *in vivo*. The *in vitro* experiments showed that PACEL and two indole analogues (Trp and pa02) had promoting HSF proliferation activity, while pa01 had an inhibiting HSF proliferation activity. Previous studies also found that extracts from PA had either promoting activity or inhibiting activity on cell proliferation (Song et al., 2017; Li et al., 2019), indicating complex components in PA may contribute to its effect on cell proliferation. In addition, the *in vivo* experiments showed that intragastric administration of PACEL, Trp, or pa02 could alleviate symptoms of weight loss and colon length shortening in the UC model mice caused by DSS, and also reduced the mortality of mice. The histological section showed tissue damages such as inflammatory cell infiltration and goblet cell loss had been alleviated after treatment with PACEL, Trp, or pa02, suggesting good healing activity and anti-inflammation activity. Although recovery of the compound pa01 on the colon length was not as obvious as other indole analogues, it showed anti-inflammatory activity in histological analysis.

Wound healing is a complex and orderly pathological process, which includes three interrelated stages: inflammation, proliferation, and remodeling. It involves the interactions between a variety of epidermal cells and fibroblasts, as well as the healing of midgut mucosal injury in UC (Diegelmann et al., 2004; Yokoyama et al., 2014; Boal Carvalho and Cotter, 2017). To reveal the molecular mechanism of the indole analogues on the recovery of UC mice, we performed qPCR to examine the effects of PACEL and indole analogues on the gene expression of two inflammatory factors (*MMP7*, *IL1α*), two intestinal mucosa-related-genes (*MUC4*, *BMP8B*), and two epithelial fibroblast related genes (*PTGS2*, *AGT*). Matrix metalloproteinase-7 gene (*MMP7*) is highly expressed in intestinal tissues of patients with enteritis, especially in intestinal epithelial cells at the edge of ulcers, so it is a common marker of inflammation (Geng, 2019). The inflammatory cytokine gene (*IL1α*) is associated with ulcerative colitis. Activation of neutrophils by *IL1α* leads to increased lysosomal enzyme activity and phagocytosis, resulting in a large number of toxic substances, thus aggravating intestinal mucosal inflammation (Muro and Mrowiec, 2015; Menghini et al., 2019). Mucin 4 gene (*MUC4*) is a member of the transmembrane mucin family, and its expression affects the viability of normal epithelial cells and the lubrication of the lumen (Das et al., 2015). The bone morphogenetic protein 8b gene (*BMP8B*) affects TGF-β signal transduction and plays an important role in maintaining intestinal mucosal immune homeostasis (Skok et al., 2021). Prostaglandin-endoperoxide synthase gene (*PTGS2*/*COX2*) is related to the occurrence of inflammation, and its coding *COX2* protein can participate in immune expression, and then affect the immune response of the body (Seton-Rogers, 2020). Angiotensinogen gene (*AGT*) is a high-risk driving gene for the occurrence and development of UC, which can activate or increase fibroblast proliferation (Garcia-Etxebarria et al., 2022). The qPCR results indicated that both PACEL and three indole analogues could alleviate DSS-induced intestinal inflammation in mice by inhibiting pro-inflammatory cytokines (*MMP7*, *IL1α*) and down-regulating *BMP8B* expression, thus preventing carcinogenesis of normal colonic epithelial cells. Moreover, they could regulate the proliferation of mouse colonic epithelial fibroblasts by up-regulating the expression of *AGT*, down-regulating *PTGS2* and *MUC4*, and alleviating the damage of the intestinal barrier induced by DSS. Thus our study highlighted that both PACEL and the three indole analogues could protect the integrity of the intestinal mucosal barrier and intestinal epithelial cells by regulating gene expression, which played a central role in repairing intestinal injury. Although several indole analogues with good anti-UC activity have been isolated in the present study, according to the HPLC analysis, it is highly likely there are other indole analogues in the PACEL. Previous studies have reported diverse pharmacological activities of PA extract, what other indole analogues in the PACEL, and how these components interact with each other contribution to the diverse bioactivities are yet to be fully revealed. Therefore, further study to comprehensively analyze the indole analogues in PA extract and elucidate their mechanisms will hold significant importance for the development of effective natural medicines.

## 5 Conclusion

As an important traditional medical insect, PA has a variety of biological and pharmacological activities. This study reported the isolation, purification, structure identification, and biological activity investigation of the main active substances in the PA extract. It was found for the first time that the extract of PA contained a large amount of Trp and indole analogues. Furthermore, based on the proliferation activity experiment *in vitro* and *in vivo* bioactivities experiments on the UC model mice, the results proved that they could be the important active components of the PA extract to promote HSF cell proliferation, wound healing, and anti-inflammation. The study has laid the foundation for further research and utilization of PA in the future.

## Data Availability

The original contributions presented in the study are included in the article/Supplementary Material, further inquiries can be directed to the corresponding authors.

## Supplementary material

SUPPLEMENTARY FIGURE S1 High resolution mass spectrometry images of pa01, pa02, pa03, and pa04.

SUPPLEMENTARY FIGURE S2 NMR spectra of pa01, pa02, pa03, and pa04 (d6-DMSO, 600 MHz).

## References

[B1] Ali S. M. Siddiqui R. Ong S. K. Shah M. R. Anwar A. Heard P. J. (2017). Identification and characterization of antibacterial compound(s) of cockroaches (*Periplaneta americana*). Appl. Microbiol. Biotechnol. 101, 253–286. 10.1007/s00253-016-7872-2 27743045

[B2] Aricioglu F. Altunbas H. (2003). Harmane induces anxiolysis and antidepressant-like effects in rats. Ann. N. Y. Acad. Sci. 1009 (1), 196–201. 10.1196/annals.1304.024 15028588

[B3] Ashok P. Chander S. Balzarini J. Pannecouque C. Murugesan S. (2015). Design, synthesis of new β-carboline derivatives and their selective anti-HIV-2 activity. Bioorg. Med. Chem. Lett. 25 (6), 1232–1235. 10.1016/j.bmcl.2015.01.058 25682562

[B4] Boal Carvalho P. Cotter J. (2017). Mucosal healing in ulcerative colitis: a comprehensive review. Drugs 77 (2), 159–173. 10.1007/s40265-016-0676-y 28078646

[B5] Bratchkova A. Ivanova V. Gousterova A. Laatsch H. (2012). β-Carboline alkaloid constituents from a Thermoactinomyces SP. strain isolated from Livingston Island, Antarctica. Biotechnol. Biotec. Eq. 26 (3), 3005–3009. 10.5504/bbeq.2012.0021

[B6] Campos Chisté R. Silva Pena R. Abreu Gloria M. B. Santos Lopes A. (2017). Bioactive amines and phenolic compounds in cocoa beans are affected by fermentation. Food Chem. 228, 484–289. 10.1016/j.foodchem.2017.02.004 28317753

[B7] Cao R. Peng W. Wang Z. Xu A. (2007). beta-Carboline alkaloids: biochemical and pharmacological functions. Curr. Med. Chem. 14 (4), 479–500. 10.2174/092986707779940998 17305548

[B8] Das S. Rachagani S. Sheinin Y. Smith L. M. Gurumurthy C. B. Roy H. K. (2015). Mice deficient in Muc4 are resistant to experimental colitis and colitis-associated colorectal cancer. Oncogene 35 (20), 2645–2654. 10.1038/onc.2015.327 26364605 PMC5555307

[B9] Di Giorgio C. Delmas F. Ollivier E. Elias R. Balansard G. Timon-David P. (2004). *In vitro* activity of the β-carboline alkaloids harmane, harmine, and harmaline toward parasites of the species *Leishmania infantum* . Exp. Parasitol. 106 (3-4), 67–74. 10.1016/j.exppara.2004.04.002 15172213

[B10] Diegelmann R. F. Evans M. C. (2004). Wound healing: an overview of acute, fibrotic and delayed healing. Front. Biosci. 9, 283. 10.2741/1184 14766366

[B11] Fu S. Chen J. Zhang C. Shi J. Nie X. Hu Y. (2021). Gastroprotective effects of *Periplaneta americana* L. extract against ethanol-Induced gastric ulcer in mice by suppressing apoptosis-related pathways. Front. Pharmacol. 12, 798421. 10.3389/fphar.2021.798421 34975497 PMC8715040

[B12] Garcia-Etxebarria K. Merino O. Gaite-Reguero A. Rodrigues P. M. Herrarte A. Etxart A. (2022). Local genetic variation of inflammatory bowel disease in Basque population and its effect in risk prediction. Sci. Rep. 12 (1), 3386. 10.1038/s41598-022-07401-2 35232999 PMC8888637

[B13] Ge H. Cai Z. Chai J. Liu J. Liu B. Yu Y. (2021). Egg white peptides ameliorate dextran sulfate sodium-induced acute colitis symptoms by inhibiting the production of pro-inflammatory cytokines and modulation of gut microbiota composition. Food Chem. 360, 129981. 10.1016/j.foodchem.2021.129981 34020366

[B14] Geng L. (2019). Expressions and significance of MMP-7 and SDC-1 in inflammatory bowel disease. Chin. J. Gastroenterology 12, 165–168.

[B15] Herraiz T. Chaparro C. (2005). Human monoamine oxidase is inhibited by tobacco smoke: β-carboline alkaloids act as potent and reversible inhibitors. Biochem. Biophys. Res. Commun. 326 (2), 378–386. 10.1016/j.bbrc.2004.11.033 15582589

[B16] Herraiz T. Galisteo J. (2002). Tetrahydro-β-carboline alkaloids that occur in foods and biological systems act as radical scavengers and antioxidants in the ABTS assay. Free Radic. Res. 36 (8), 923–928. 10.1080/1071576021000005762 12420751

[B17] Herraiz T. (2007). Identification and occurrence of beta-carboline alkaloids in raisins and inhibition of monoamine oxidase (MAO). J. Agric. Food Chem. 55 (21), 8534–8540. 10.1021/jf0719151 17883257

[B18] Innocente N. Biasutti M. Padovese M. Moret S. (2007). Determination of biogenic amines in cheese using HPLC technique and direct derivatization of acid extract. Food Chem. 101 (3), 1285–1289. 10.1016/j.foodchem.2005.12.026

[B19] Khan M. Z. Nawaz W. (2016). The emerging roles of human trace amines and human trace amine-associated receptors (hTAARs) in central nervous system. Biomed. Pharmacother. 83, 439–449. 10.1016/j.biopha.2016.07.002 27424325

[B20] Kim I. W. Lee J. H. Subramaniyam S. Yun E. Y. Kim I. Park J. (2016). *De novo* transcriptome analysis and detection of antimicrobial peptides of the American cockroach *Periplaneta americana* (Linnaeus). PLoS One 11 (5), e0155304. 10.1371/journal.pone.0155304 27167617 PMC4864078

[B21] Kim J. J. Shajib M. S. Manocha M. M. Khan W. I. (2012). Investigating intestinal inflammation in DSS-induced model of IBD. J. Vis. Exp. 60, e3678. 10.3791/3678 PMC3369627 22331082

[B22] Li C. Ai G. Wang Y. Lu Q. Luo C. Tan L. (2020). Oxyberberine, a novel gut microbiota-mediated metabolite of berberine, possesses superior anti-colitis effect: impact on intestinal epithelial barrier, gut microbiota profile and TLR4-MyD88-NF-κB pathway. Pharmacol. Res. 152, 104603. 10.1016/j.phrs.2019.104603 31863867

[B23] Li D. Li W. Chen Y. Liu L. Ma D. Wang H. (2018). Anti-fibrotic role and mechanism of *Periplaneta americana* extracts in CCl4-induced hepatic fibrosis in rats. Acta Biochim. Biophys. Sin. (Shanghai) 50 (5), 491–498. 10.1093/abbs/gmy024 29538616 PMC5946930

[B24] Li L. J. Xu X. H. Yuan T. J. Hou J. Yu C. L. Peng L. H. (2019). *Periplaneta Americana* L. as a novel therapeutics accelerates wound repair and regeneration. Biomed. Pharmacother. 114, 108858. 10.1016/j.biopha.2019.108858 30986622

[B25] Li Y. Wang F. Zhang P. Z. Yang M. (2015). Chemical constituents from *Periplaneta americana* . J. Chin. Med. Mater. 38 (10), 2038–2041. 27254913

[B26] Li Y. Wang X. Su Y. Wang Q. Huang S. Pan Z. (2021). Baicalein ameliorates ulcerative colitis by improving intestinal epithelial barrier via AhR/IL-22 pathway in ILC3s. Acta Pharmacol. Sin. 43, 1495–1507. 10.1038/s41401-021-00781-7 34671110 PMC9160000

[B27] Liang Z. Han G. Luo Z. Li B. Li W. Shen C. (2022). Effects of *Periplaneta americana* extracts on the growth and proliferation of cutaneous interstitial cells in cutaneous-wound healing. Front. Pharmacol. 13, 920855. 10.3389/fphar.2022.920855 36105218 PMC9465176

[B28] Liu Y. Li B. G. Su Y. H. Zhao R. X. Song P. Li H. (2022). Potential activity of traditional Chinese medicine against ulcerative colitis: a review. J. Ethnopharmacol. 289, 115084. 10.1016/j.jep.2022.115084 35134488

[B29] Lu S. Wu D. Sun G. Geng F. Shen Y. Tan J. (2019). Gastroprotective effects of Kangfuxin against water-immersion and restraint stress-induced gastric ulcer in rats: roles of antioxidation, anti-inflammation, and pro-survival. Pharm. Biol. 57 (1), 770–777. 10.1080/13880209.2019.1682620 31696757 PMC6844415

[B30] Machado M. W. Stern Neto C. Salgado J. Zaffari G. Barison A. Campos F. R. (2010). Search for alkaloids on callus culture of *Passiflora alata* . Braz. Arch. Biol. Technol. 53 (4), 901–910. 10.1590/s1516-89132010000400020

[B31] Menghini P. Corridoni D. Buttó L. F. Osme A. Shivaswamy S. Lam M. (2019). Neutralization of IL-1α ameliorates Crohn's disease-like ileitis by functional alterations of the gut microbiome. Proc. Natl. Acad. Sci. U.S.A. 116 (52), 26717–26726. 10.1073/pnas.1915043116 31843928 PMC6936591

[B32] Miller K. D. Siegel R. L. Lin C. C. Mariotto A. B. Kramer J. L. Rowland J. H. (2016). Cancer treatment and survivorship statistics, 2016. CA Cancer J. Clin. 66 (4), 271–289. 10.3322/caac.21349 27253694

[B33] Muro M. Mrowiec A. (2015). Interleukin (IL)-1 gene cluster in inflammatory bowel disease: is IL-1RA implicated in the disease onset and outcome? Dig. Dis. Sci. 60 (5), 1126–1128. 10.1007/s10620-015-3571-6 25875754

[B34] Peng C. Wu C. Xu X. Pan L. Lou Z. Zhao Y. (2021). Indole-3-carbinol ameliorates necroptosis and inflammation of intestinal epithelial cells in mice with ulcerative colitis by activating aryl hydrocarbon receptor. Exp. Cell. Res. 404, 112638. 10.1016/j.yexcr.2021.112638 34015312

[B35] Sarvestani S. K. Signs S. Hu B. Yeu Y. Feng H. Ni Y. (2021). Induced organoids derived from patients with ulcerative colitis recapitulate colitic reactivity. Nat. Commun. 12 (1), 262. 10.1038/s41467-020-20351-5 33431859 PMC7801686

[B36] Seton-Rogers S. (2020). Fibroblasts orchestrate tumour initiation. Nat. Rev. Cancer 20 (6), 301. 10.1038/s41568-020-0264-z 32317743

[B37] Shi C. C. Liao J. F. Chen C. F. (2001). Comparative study on the vasorelaxant effects of three harmala alkaloids *in vitro* . Jpn. J. Pharmacol. 85 (3), 299–305. 10.1254/jjp.85.299 11325023

[B38] Shi J. Guo S. Wu Y. Chen G. Lai J. Xu X. (2019). Behaviour of cell penetrating peptide TAT-modified liposomes loaded with salvianolic acid B on the migration, proliferation, and survival of human skin fibroblasts. J. Liposome Res. 30 (1), 93–106. 10.1080/08982104.2019.1593451 31012367

[B39] Skok D. J. Hauptman N. Jerala M. Zidar N. (2021). Expression of cytokine-coding genes *BMP8B, LEFTY1* and *INSL5* could distinguish between ulcerative colitis and crohn’s disease. Genes. 12 (10), 1477. 10.3390/genes12101477 34680872 PMC8535226

[B40] Song Q. Xie Y. Gou Q. Guo X. Yao Q. Gou X. (2017). JAK/STAT3 and Smad3 activities are required for the wound healing properties of *Periplaneta americana* extracts. Int. J. Mol. Med. 40 (2), 465–473. 10.3892/ijmm.2017.3040 28656220 PMC5504994

[B41] Suzuki K. Nomura I. Ninomiya M. Tanaka K. Koketsu M. (2018). Synthesis and antimicrobial activity of β-carboline derivatives with N2-alkyl modifications. Bioorg. Med. Chem. Lett. 28 (17), 2976–2978. 10.1016/j.bmcl.2018.06.050 30001916

[B42] Vikström Bergander L. Cai W. Klocke B. Seifert M. Pongratz I. (2012). Tryptamine serves as a proligand of the AhR transcriptional pathway whose activation is dependent of monoamine oxidases. Mol. Endocrinol. 26 (9), 1542–1551. 10.1210/me.2011-1351 22865928 PMC5416975

[B43] Xiu F. Li X. Zhang W. He F. Ying X. Stien D. (2018). A new alkaloid from *Portulaca oleracea* L. and its antiacetylcholinesterase activity. Nat. Prod. Res. 33 (18), 2583–2590. 10.1080/14786419.2018.1460833 29665731

[B44] Yang Z. Xie J. Huang F. Yang Y. Zhang X. Yue B. (2022). GC-MS analysis of chemical constituents and determination of the total antioxidant capacity of adult powder of *Periplaneta americana* . Entomol. Res. 52 (2), 68–76. 10.1016/j.jcis.2022.06.132

[B45] Yao K. Fang J. Yin Y. L. Feng Z. M. Tang Z. R. Wu G. (2011). Tryptophan metabolism in animals important roles in nutrition and health. Front. Biosci. Sch. Ed. 3 (1), 286–297. 10.2741/s152 21196377

[B46] Ye L. Jia Y. Ji K. E. Sanders A. J. Xue K. Ji J. (2015). Traditional Chinese medicine in the prevention and treatment of cancer and cancer metastasis. Oncol. Lett. 10 (3), 1240–1250. 10.3892/ol.2015.3459 26622657 PMC4533180

[B47] Yokoyama Y. Matsuoka K. Kobayashi T. Sawada K. Fujiyoshi T. Ando T. (2014). A large-scale, prospective, observational study of leukocytapheresis for ulcerative colitis: treatment outcomes of 847 patients in clinical practice. J. Crohns Colitis 8 (9), 981–991. 10.1016/j.crohns.2014.01.027 24556083

[B48] Yuan Y. Win Aung K. K. Ran X. K. Wang X. T. Dou D. Q. Dong F. (2017). A new sesquiterpene lactone from yacon leaves. Nat. Prod. Res. 31 (1), 43–49. 10.1080/14786419.2016.1212028 27484732

[B49] Zeng C. Liao Q. Hu Y. Shen Y. Geng F. Chen L. (2019). The role of *Periplaneta americana* (Blattodea: blattidae) in modern versus traditional Chinese medicine. J. Med. Entomol. 56 (6), 1522–1526. 10.1093/jme/tjz081 31265723

[B50] Zhang X. F. Sun R. Q. Jia Y. F. Chen Q. Tu R. F. Li K. K. (2016). Synthesis and mechanisms of action of novel harmine derivatives as potential antitumor agents. Sci. Rep. 6, 33204. 10.1038/srep33204 27625151 PMC5021947

[B51] Zhang Y. J. Zhang Y. Zhou Y. Li G. H. Yang W. Z. Feng X. S. (2019). A review of pretreatment and analytical methods of biogenic amines in food and biological samples since 2010. J. Chromatogr. A 1605, 360361. 10.1016/j.chroma.2019.07.015 31327479

[B52] Zhao B. Zhang Y. Xu J. Li Y. Yuan Q. Zhou C. (2022). *Periplaneta Americana* extract inhibits osteoclastic differentiation *in vitro* . Cell. Prolif. 56 (2), e13341. 10.1111/cpr.13341 36210640 PMC9890529

[B53] Zhao Y. Yang A. Tu P. Hu Z. (2017). Anti-tumor effects of the American cockroach, *Periplaneta americana* . Chin. Med. 12 (1), 26. 10.1186/s13020-017-0149-6 28919922 PMC5596864

[B54] Zhu J. J. Yao S. Guo X. Yue B. S. Ma X. Y. Li J. (2018). Bioactivity-guided screening of wound-healing active constituents from American cockroach (*Periplaneta americana*). Molecules 23 (1), 101. 10.3390/molecules23010101 29361715 PMC6017267

