# Supplementary figures and images for: Discovery of indole analogues from Periplaneta americana extract and their activities on cell proliferation and recovery of ulcerative colitis in mice
Source: Front Pharmacol. 2023 Oct 20;14:1282545. doi: 10.3389/fphar.2023.1282545 (PMC10623332; doi:10.3389/fphar.2023.1282545)

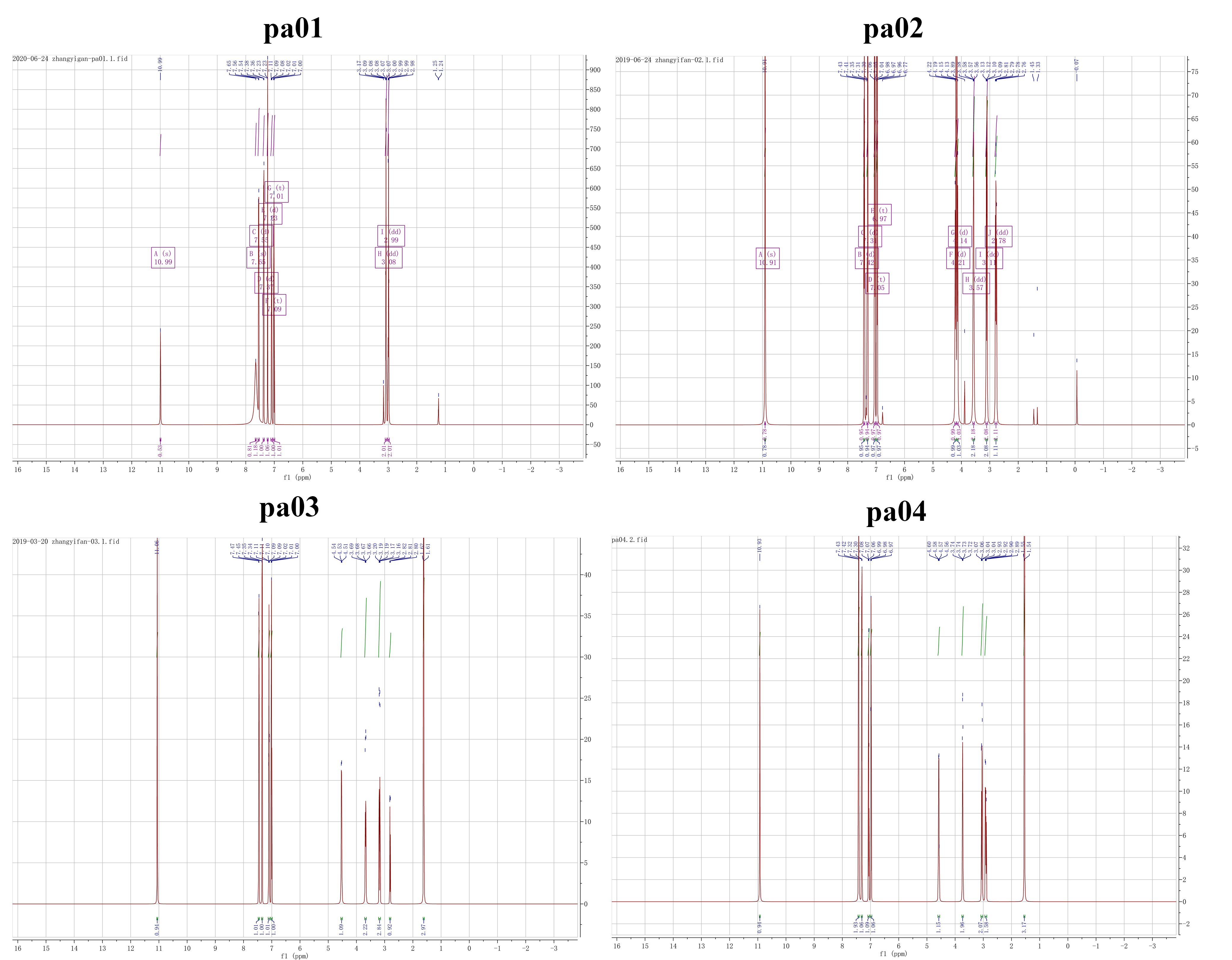

Supplement: Supplementary file 1 [file Image2.PNG]

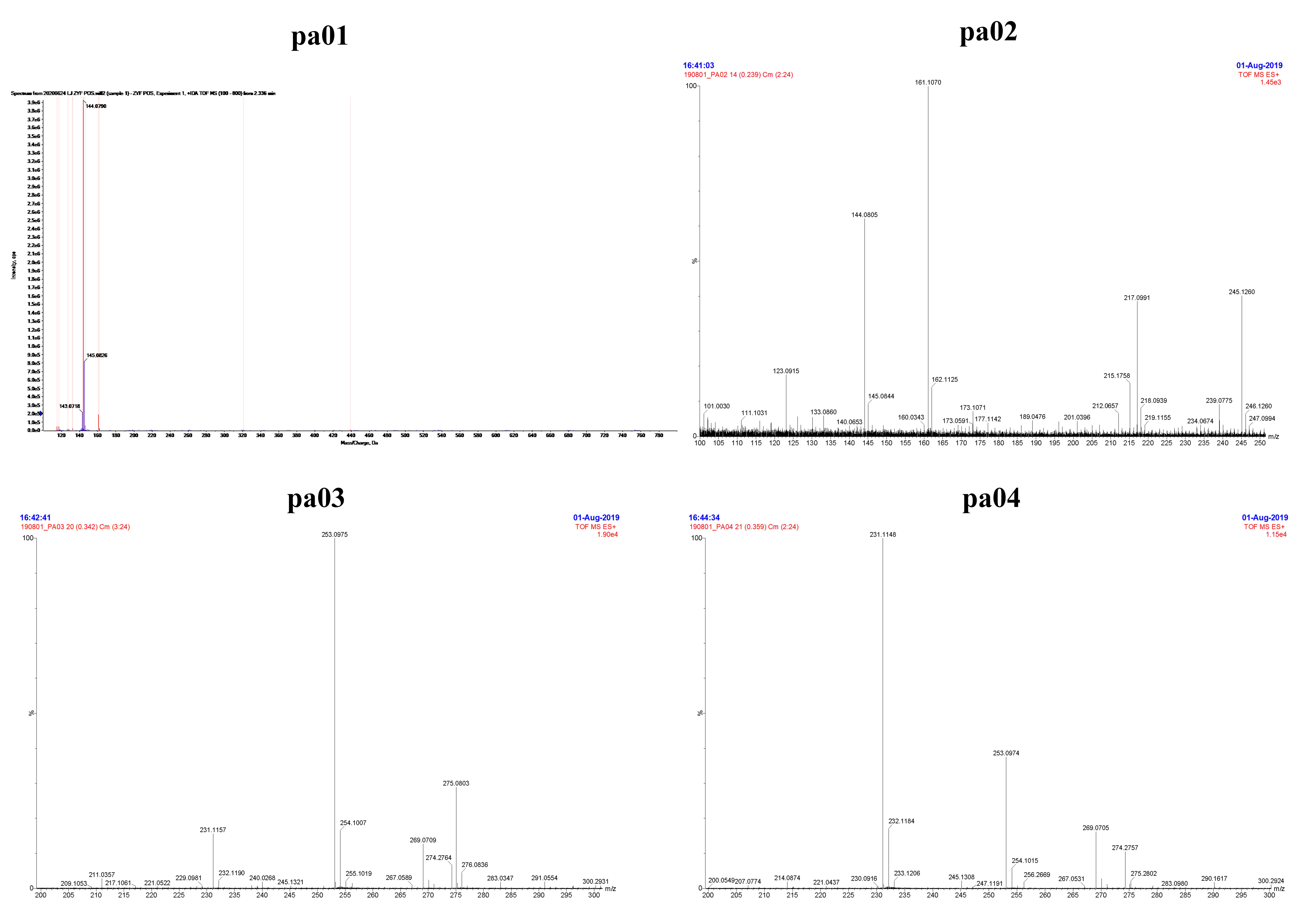

Supplement: Supplementary file 2 [file Image1.PNG]
